# Loss of Glycine *N*-Methyltransferase Associates with Angiopoietin-Like Protein 8 Expression in High Fat-Diet-Fed Mice

**DOI:** 10.3390/ijms20174223

**Published:** 2019-08-29

**Authors:** Jian-Wei Huang, Chao-Ju Chen, Chia-Hung Yen, Yi-Ming Arthur Chen, Yu-Peng Liu

**Affiliations:** 1 Graduate Institute of Medicine, Kaohsiung Medical University, Kaohsiung 80708, Taiwan; 2 Division of General Surgery, Department of Surgery, Kaohsiung Medical University Hospital, Kaohsiung Medical University, Kaohsiung 80708, Taiwan; 3 Department of Laboratory Medicine, Kaohsiung Medical University Hospital, Kaohsiung Medical University, Kaohsiung 80708, Taiwan; 4 Graduate Institute of Natural Products, College of Pharmacy, Kaohsiung Medical University, Kaohsiung 80708, Taiwan; 5 Department of Medical Research, Kaohsiung Medical University Hospital, Kaohsiung Medical University, Kaohsiung 80708, Taiwan; 6 Master Program of Clinical Pharmacogenomics and Pharmacoproteomics, College of Pharmacy, Taipei Medical University, Taipei 11031, Taiwan; 7 Graduate Institute of Clinical Medicine, Kaohsiung Medical University, Kaohsiung 80708, Taiwan; 8 Research Center for Environmental Medicine, Kaohsiung Medical University, Kaohsiung 80708, Taiwan

**Keywords:** glycine *N*-methyltransferase, Angiopoietin-like protein 8, metabolic syndrome, high fat diet, insulin

## Abstract

Imbalance of lipid metabolism is a main cause of metabolic syndrome leading to life-threatening metabolic diseases. Angiopoietin-like protein 8 (Angptl8) was recently identified as a liver and adipose tissue-released hormone that is one of the molecules involved in triglyceride metabolism. However, the regulatory mechanism of Angptl8 is largely unknown. A high fat diet (HFD)-fed mouse model, which showed high cholesterol, high triglyceride, and high insulin in the blood, revealed the upregulation of hepatic and plasma Angptl8 and the downregulation of hepatic glycine *N*-methyltransferase (GNMT). The inverse correlation of hepatic Angptl8 and GNMT expression in the livers of HFD-fed mice was also confirmed in a publicly available microarray dataset. The mechanistic study using primary hepatocytes showed that the Angptl8 expression could be induced by insulin treatment in a dose- and time-dependent manner. Inhibition of PI3K/Akt pathway by the specific inhibitors or the dominant-negative Akt blocked the insulin-induced Angptl8 expression. Moreover, knockout of GNMT promoted the Akt activation as well as the Angptl8 expression. These results suggested that GNMT might be involved in insulin-induced Angptl8 expression in HFD-mediated metabolic syndrome.

## 1. Introduction

Obesity and metabolic syndrome due to over-nutrition and sedentary lifestyle are the risk factors for nonalcoholic fatty liver disease, cardiovascular disease, and type 2 diabetes [1,2]. In recent years, more attention has been paid to the proteins released from muscle, adipose, and liver tissues that affect lipid and glucose metabolism systematically [3,4,5]. The metabolism-related organokines include proinflammatory cytokines, hormones, and growth factors [6,7,8]. The crucial pathogenic roles of some adipokines, myokines, and hepatokines have been proven in a broad range of metabolic diseases [9,10]. Given the increasing prevalence of obesity and diabetes in the modern world, it is urgent to identify new insights into the molecular basis of diet-induced metabolic disorders.

Angptl8, also known as Betatrophin/TD26/Lipasin/RIFL/C19orf80, is a 22-kDa protein with 198 amino acids. Angptl8 protein contains an N-terminal coiled-coil domain, which is shared by all members of ANGPTL family and is required for the inhibition of lipoprotein lipase activity. Unlike other members, Angptl8 lacks the C-terminal fibrinogen-like domain [11]. It has been identified as a hormone that is produced and released from liver and adipose tissues [12]. Angptl8 functions as a lipogenic factor that regulates triglyceride metabolism [13]. Mice lacking Angptl8 have lower triglyceride levels in their blood [14]. The blockage of plasma Angptl8 by a specific monoclonal antibody promotes triglyceride clearance and weight loss of the antibody-treated mice [15]. In high-fat diet (HFD)-fed mice, the hepatic Angltl8 levels were increased [16]. In the clinical view of Angptl8 regulation, elevation of circulating Angptl8 was associated with polycystic ovary syndrome [17], hypertension [18], obesity, and type 2 diabetes [19]. In fact, losing weight by a calorie-restricted diet and activity decreases the plasma levels of Angptl8 [20,21]. These findings from animal and clinical studies suggested that dysregulation of Angptl8 can be the cause and consequence of a metabolic syndrome.

Although Angptl8 expression has been strongly linked to the onset of metabolic syndrome, the molecular regulation of Angptl8 is largely unknown. It has been shown that Angptl8 expression can be inhibited by microRNAs [22,23]. In addition, the expression of Angptl8 in liver can be regulated through insulin-mediated PI3K/Akt [24], as well as liver X receptor alpha (LXRα)- and glucocorticoid receptor-induced signaling pathways [25]. However, the regulation of Angptl8 expression in pathological conditions is still unclear. As the deregulation of several transcription factors, cytokine, and enzymes in liver is the hallmark of metabolic syndrome, we hypothesized that Angptl8 could be regulated by liver-derived and metabolism-related factors.

Glycine *N*-methyltransferase (GNMT) is an enzyme that catalyzes the conversion of hepatic s-adenosylmethionine (SAM) to s-adenosylhomocysteine (SAH). Accumulation of SAM and downregulation of GNMT are frequently found in rodent models of HFD-induced nonalcoholic fatty liver disease, [26]. It has been shown that GNMT-deficient mice developed chronic hepatitis, steatosis, and hepatocellular carcinoma (HCC) [27,28]. Recently, downregulation of GNMT has been considered as a hallmark of hepatocellular carcinoma (HCC) [29]. In addition to its metabolic function, GNMT has been identified as a signaling molecule by promoting the protein HectH9-dependent degradation of PREX2 [30], an inhibitor of PTEN [31]. Since PTEN is an inhibitor of PI3K/Akt signaling cascade, depletion of GNMT results in the activation of Akt. 

Since GNMT and Angptl8 play an important role in lipid metabolism in physiological and pathological conditions, we hypothesized that GNMT may involve in Angptl8 expression. In this study, we demonstrated an inverse correlation of Angptl8 and GNMT expression from a HFD-fed mouse model and a publicly available microarray dataset. The mechanistic study, using primary mouse hepatocytes, showed that Angptl8 expression was induced by the insulin-induced PI3K/Akt signaling pathway. Furthermore, deficiency of GNMT induced Angptl8 expression via the PI3K/Akt signaling pathway. Our data suggested that the GNMT may function as a negative regulator for Angptl8 expression in metabolic syndrome.

## 2. Results

### 2.1. Increase of Plasma Insulin in High-Fat Diet Mice

**Figure 1:**
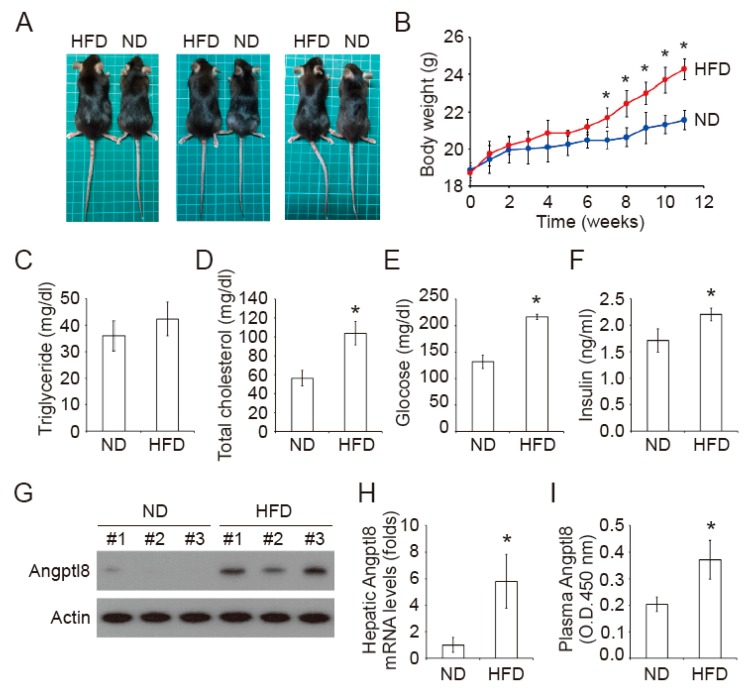
Characterization of the mice fed with HFD and ND. (**A**) The representative pictures showing HFD-fed and ND-fed mice. (**B**) The body weight of the mice was measured weekly; * *p* < 0.05, compared to the ND control group. (**C**–**F**) The fasting triglyceride (**C**), total cholesterol (**D**), glucose (**E**), and insulin (**F**) in the blood of the mice were analyzed; * *p* < 0.05, compared to the relative ND control groups. (**G**) The hepatic Angptl8 expression was examined by Western blot. (**H**) The mRNA levels of hepatic Angptl8 was analyzed by qPCR; * *p* < 0.05, compared to the ND control group. (**I**) The plasma Angptl8 was analyzed by ELISA; * *p* < 0.05, compared to the ND control group.

To access the regulation of Angptl8 in metabolic syndrome, we first established a HFD-induced obese mouse model. The mice were fed with normal diet (ND) or HFD for 12 weeks. The food intake in a HFD group and a ND group was not different (Table 1). However, the body weight, body mass index (BMI), and food efficiency ratio (FER) were higher (*p* < 0.05) in a HFD group compared to these in a ND group (Table 1, Figure 1A,B). The liver weight and hepatosomatic index (HSI) of mice in the HFD group was significantly higher than that of mice in the ND group (Table 1). After a 12 week feeding of ND or HFD, the fasting blood triglyceride (TG) levels of the mice in the HFD group did not differ from the ND group (Figure 1C). However, the fasting blood glucose and total cholesterol levels of mice in the HFD group were higher than those of mic in the ND group (Figure 1D,E). The steady-state blood insulin was increased in the HFD group compared to that in the ND group (Figure 1F). On the other hand, the hepatic Angptl8 protein and mRNA levels were elevated in the livers of HFD-fed mice compared to its levels in the livers of ND-fed mice (Figure 1G,H). In parallel, the plasma Angptl8 levels were increased in the HFD-fed mice compared to the ND-fed mice (Figure 1I).

### 2.2. Insulin Induces Akt Phosphorylation and Angptl8 Expression

**Figure 2:**
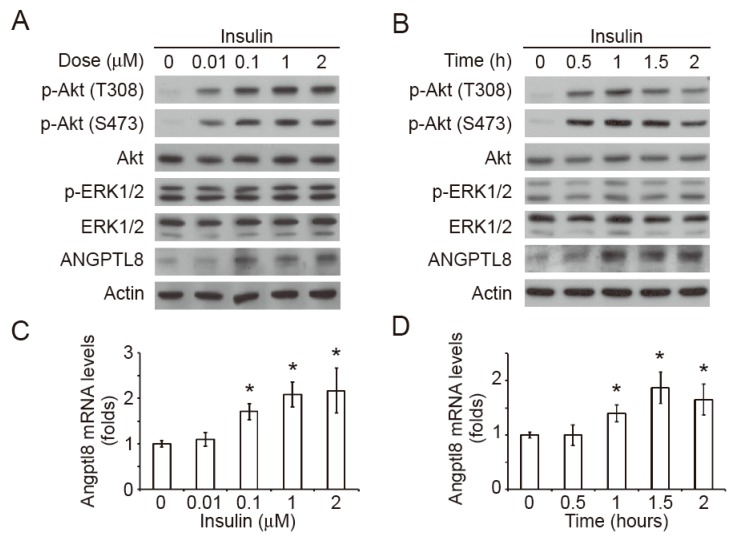
Insulin induces Akt phosphorylation and Angptl8 expression. (**A**) The primary hepatocytes were treated with insulin at different concentrations for 1 h. The expression of indicated proteins was examined by Western blot. (**B**) The primary hepatocytes were treated with insulin (1 μM) for different time periods. The expression of the indicated proteins was examined by Western blot. (**C**–**D**) The hepatic Angptl8 mRNA levels in the primary hepatocytes treated with or without insulin at different concentrations (**C**) and time periods (**D**) were analyzed by qPCR; * *p* < 0.05, compared to the control group.

Since plasma insulin was increased in the HFD-fed mice, we hypothesized that insulin-mediated signaling pathways could be the upstream regulators of *Angptl8*. Accordingly, we isolated primary hepatocytes from the livers of ND-fed C57BL/6J mice. The primary hepatocytes were treated with different concentrations of insulin for 1 h. The data showed that the insulin treatment induced Angptl8 expression and phosphorylation of Akt at Threonine 308 (T308) and Serine 473 (S473) residues and in a dose-dependent manner (Figure 2A). However, the insulin treatment did not alter the phosphorylation of ERK1/2. On the other hand, the treatment of 1 M insulin time-dependently increased Angptl8 expression within 1 h and the levels of Angptl8 reached a plateau after 1 h of insulin treatment (Figure 2B). The insulin-induced Akt phosphorylation at T308 and S473 reached a peak after 1 h of the treatment. Similarly, the phosphorylation of ERK1/2 was not changed during the periods of insulin treatment. The results from qPCR showed that the insulin treatment induced a dose- and time-dependent upregulation of Angptl8 mRNA expression (Figure 2C,D).

### 2.3. Insulin-Induced Angptl8 is Mediated by PI3K/Akt Signaling Pathway

**Figure 3:**
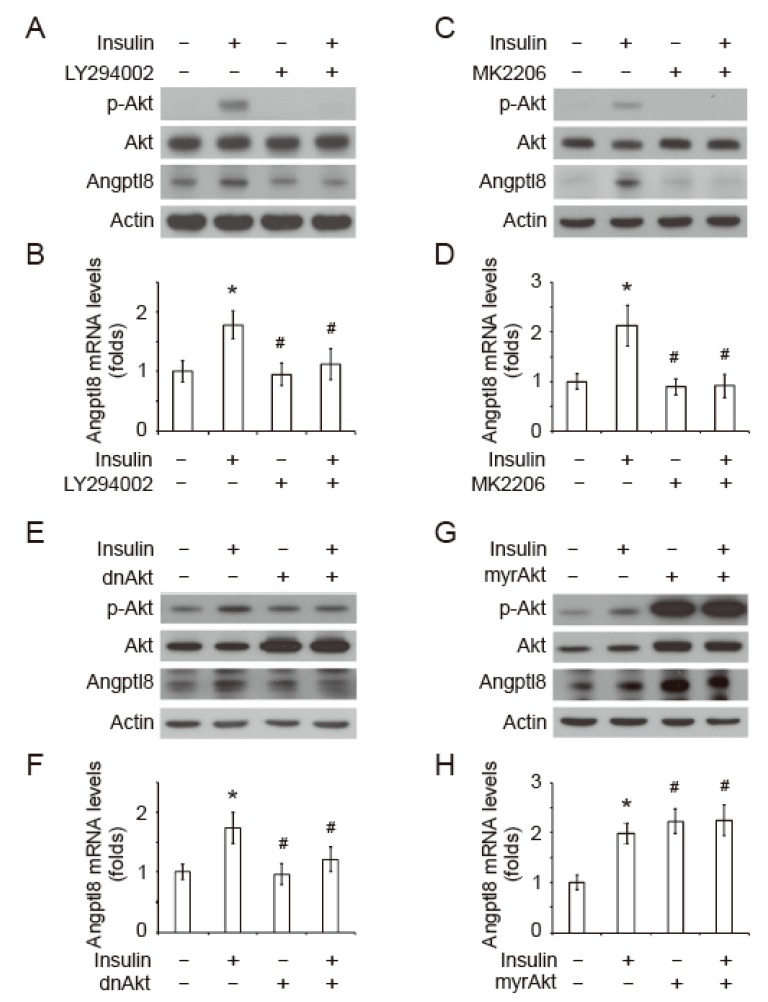
Insulin-induced Angptl8 is mediated by the PI3K/Akt signaling pathway. (**A**–**B**) The primary hepatocytes were incubated in the culture medium with or without insulin (1 μM) and LY294002 (10 μM) for 1 h. The expression of indicated proteins (**A**) and Angptl8 mRNA (**B**) was examined by Western blot and qPCR respectively. (**C**–**D**) The primary hepatocytes were incubated in the culture medium with or without insulin (1 μM) and MK2206 (10 μM) for 1 h. The expression of indicated proteins (**C**) and Angptl8 mRNA (**D**) was examined by Western blot and qPCR, respectively. (**E**–**F**) The primary hepatocytes were transiently transfected with dominant-negative Akt plasmids, followed by an insulin treatment for 1 h. The expression of indicated proteins (**E**) and Angptl8 mRNA (**F**) was examined by Western blot and qPCR, respectively. (**G**–**H**) The primary hepatocytes were transiently transfected with constitutively activated myrAkt plasmids followed by a treatment of insulin for 1 h. The expression of indicated proteins (**G**) and Angptl8 mRNA (**H**) was examined by Western blot and qPCR, respectively.

To investigate whether Angptl8 expression can be regulated by insulin-induced Akt signaling pathway, the primary hepatocytes were pre-treated with a PI3K inhibitor, LY294002, for 20 min, followed by a treatment of 1 μM insulin for 1 h. The results showed that the pre-treatment of LY294002 inhibited insulin-induced Akt phosphorylation at T308, as well as insulin-induced Angptl8 protein and mRNA expression (Figure 3A,B). In parallel, the insulin-induced Angptl8 protein and mRNA expression was inhibited by a selective Akt inhibitor, MK2206 (Figure 3C,D). To further confirm the role of Akt signaling on Angptl8 expression, a dominate-negative Akt plasmid (dnAkt), a kinase-dead form of Akt, or an empty vector was transfected into the primary hepatocytes, followed by the treatments of insulin. The results showed that the cells expressing dnAkt significantly inhibited the protein and mRNA expression of Angptl8 (Figure 3E,F). Furthermore, we next studied whether the activation of Akt is sufficient to elevate Angptl8 expression. Ectopic expression of a constitutively active form of Akt, myrAkt, significantly increased Angptl8 expression with or without insulin treatment. These results demonstrated that the insulin-induced PI3K-Akt signaling pathway is an upstream regulator of Angptl8.

### 2.4. GNMT/PI3K/Akt Signaling Cascade Regulates Angptl8 Expression

**Figure 4:**
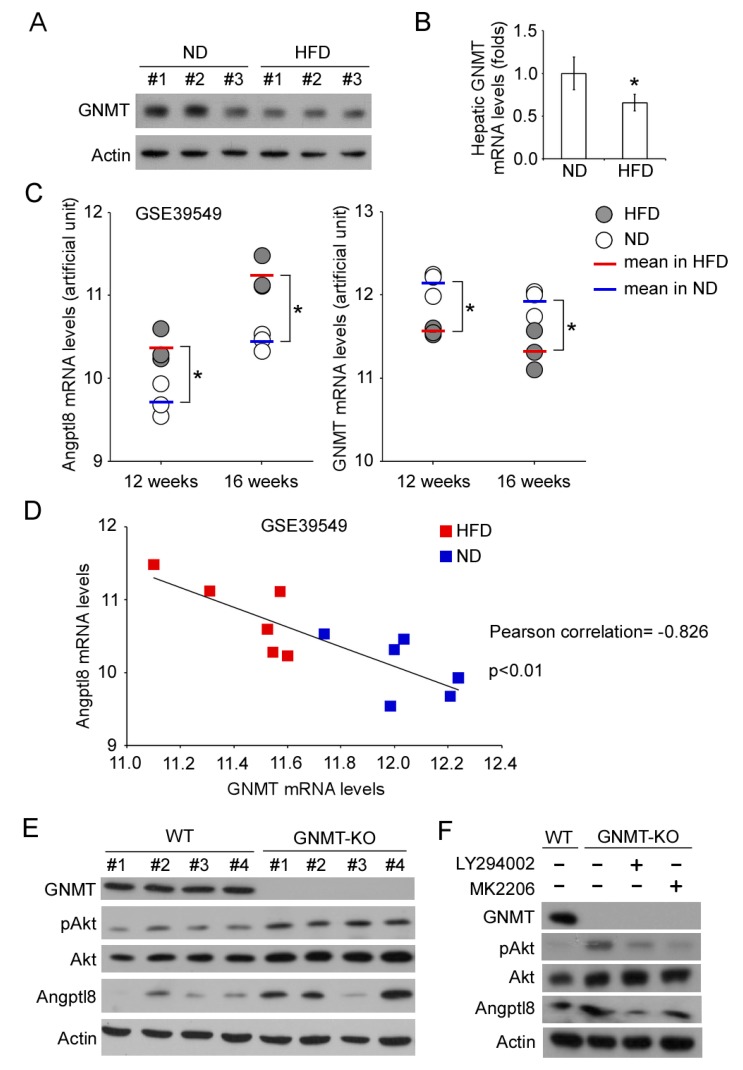
The inverse correlation of GNMT and Angptl8 expression in the livers of HFD-fed mice. (**A**–**B**) The expression of hepatic GNMT protein (**A**) and mRNA (**B**) in the HFD-fed and ND-fed mice was revealed by Western blot and qPCR respectively. (**C**) The data of Angptl8 and GNMT mRNA expression was extracted from a microarray dataset, GSE39549; * *p* < 0.05, compared between the HFD and ND groups. (**D**) The data of Angptl8 and GNMT mRNA levels for each mouse were plotted. The inverse correlation of Angptl8 and GNMT was confirmed by Pearson’s correlation; * *p* < 0.01. (**E**) The liver tissues were obtained from wild type and GNMT-KO mice. The expression of indicated proteins were examined by Western blot. (**F**) The primary culture of hepatocytes was established from wild type and GNMT-KO mice. The primary hepatocytes were treated with LY294002 or MK2206, and the expression of indicated proteins was determined by Western blot.

To investigate whether GNMT may be involved in Angptl8 regulation, we first analyzed the GNMT expression in HFD-fed mice. The hepatic GNMT protein levels of mice in a HFD group were downregulated compared to that of mice in a ND group (Figure 4A). The GNMT mRNA levels were also significantly decreased in the livers of HFD-fed mice compared to the mice in a ND group (Figure 4B). To further confirm the relationship between GNMT and Angptl8 expression, we analyzed the mRNA levels of GNMT and Angptl8 in a publicly available microarray dataset, GSE39549, see References [32,33]. In this microarray dataset, the C57BL/6J mice were fed with ND or HFD for multiple time periods and the adipose tissues of mice were subjected to microarray analysis. The Angptl8 mRNA expression in the adipose tissues of the HFD-fed mice was higher than that of the ND-fed mice (*p* < 0.05), while GNMT mRNA expression was downregulated in the adipose tissues of the HFD-fed mice (*p* < 0.05) (Figure 4C). Pearson’s correlation showed that the Angptl8 mRNA expression inversely correlated with the GNMT mRNA expression (*p* < 0.01) (Figure 4D). In the livers of GNMT-knockout (GNMT-KO) mice, total Akt and phosphorylated Akt at T308 was increased and the hepatic Angptl8 protein levels were also upregulated compared to that in the livers of wild-type mice (Figure 4E). These results indicated that GNMT might be an upstream regulator of Angptl8. In the primary hepatocytes isolated from GNMT-KO mice, LY294003 and MK2206 still inhibited that Angptl8 expression (Figure 4F). These results suggested a GNMT/PI3K/Akt signaling cascade that can regulate Angptl8 expression.

## 3. Discussion

Elevation of Angptl8 levels in circulation has been recently considered as a potential biomarker for metabolic diseases [34]. This phenomenon could be observed in multiple animal models, such as non-alcoholic fatty liver disease and non-alcoholic fatty liver disease of HFD or methionine-choline deficient (MCD) diet-fed mice [35], as well as obesity-related syndrome of ob/ob or db/db mice [16]. In a physiological condition, e.g., in normal diet-fed mice, hepatic Angptl8 levels are rhythmically expressed under the regulation of glucocorticoid signaling and the LXRα pathway [25]. A recent study also showed that the Angptl8 mediated food-driven resetting of hepatic circadian clock [36]. In our study, the increase of total cholesterol, insulin, and glucose in the blood of HFD-fed mice indicated a metabolic syndrome in the mice. In agreement with the findings from others, the hepatic and plasma Angptl8 was increased in the HFD-fed mice. We therefore hypothesized that plasma insulin might be one of the factors that promotes Angptl8 production. Indeed, our in vitro study using primary hepatocytes demonstrated that the insulin-activated PI3K/Akt signaling pathway significantly induced Angptl8 expression in a dose and time-dependent manner. Blockage of the PI3K/Akt signaling cascade using the PI3K and Akt inhibitors, as well as the dominant-negative Akt, inhibited Angptl8 expression. For an unknown reason, we found the treatment of insulin resulted in the phosphorylation of Akt, but did not alter the phosphorylation levels of ERK1/2 in the primary hepatocytes. Further experimentation is required to study the protein switches that may control the differential activation of PI3K/Akt and ERK1/2 signaling upon insulin stimulation in primary hepatocytes. Our data indicated that elevation of plasma insulin is an important factor that induces the upregulation of hepatic Angptl8 expression. Consistent with this result, a previous study showed that the expression of Angptl8 was only upregulated in the liver, white adipose tissue and brown adipose tissue of obesity-induced hyperinsulinemic type 2 diabetes mice, but it was decreased in white adipose tissue of streptozotocin-induced hypoinsulinemic type 1 diabetes mice [37]. Together, our data and the results from other studies show that insulin-mediated PI3K/Akt signaling pathway is a major upstream regulator for HFD-induced Angptl8 expression, but not blood glucose. However, adipose tissue and muscle also act as endocrine organs that release various cytokines, such as leptin, adiponectin, tumor necrosis factor-alpha, and interleukin-6, and alter insulin sensitivity in obesity and metabolic syndrome [38]. A recent study showed that the Angptl8 expression was associated with hyperactive catabolism of the extracellular matrix (ECM) and inflammation [39]. Accordingly, we cannot exclude other factors, such as proinflammatory cytokines, may also be involved in HFD-induced Angptl8 regulation. Further experiments are required to investigate the effects of liver-, adipose tissue-, and muscle-derived cytokines in Angptl8 expression.

All angiopoietin-like proteins (Angptl1–8) are secretory glycoproteins. Among the family members, Angptl3, Angptl4, and Angptl8 are involved in lipoprotein metabolism by governing lipoprotein lipase activity [40]. Structurally, they share an N-terminal coiled-coil domain, which is required for the inhibition of lipoprotein lipase activity. Unlike Angptl3 and Angptl4, Angptl8 lacks a C-terminal fibrinogen-like domain, which has antiangiogenic functions [11]. Functionally, overexpression of hepatic Angptl8 elevated plasma triglyceride levels in wild type mice, but not in Angptl3-KO mice [12,14], suggesting that Angptl3 and Angptl8 work together to regulate triglyceride metabolism. However, Angptl4 plays a distinct function rather than Angptl3 and Angptl8. Angptl3 and Angptl8 promotes the post-prandial flux of triglyceride into adipose tissue in fed state, while Angptl4 prevents the uptake of circulating triglyceride into adipose tissue in fasted state [41,42]. Accordingly, elevation of Angptl8 may not be the only cause that increases plasma triglyceride. More studies are required to elucidate the regulatory network of angiopoietin-like proteins in pathological conditions.

In liver, GNMT is an abundant enzyme that contributes to the conversion of SAM, which is the methyl donor required for the methylation of phosphatidylethanolamine (PE). PE can be converted into phosphatidylcholine (PC) through the phosphatidylethanolamine *N*-methyltransferase (PEMT) pathway. Accumulation of hepatic SAM and PC is found in the livers of GNMT-KO mice [43]. The excess of SAM and PC contribute to the triglyceride synthesis and release. Indeed, the increase of hepatic SAM and the decrease of hepatic GNMT were linked to non-alcoholic fatty liver disease in a HFD-fed rodent model [44]. The down-regulation of GNMT can be through the DNA methylation of its promoter region and microRNA-mediated post-transcriptional regulation in disease models and clinical studies, liver fibrosis and cirrhosis, and HCC [45,46]. In our study, we showed that downregulation of GNMT might contribute to the elevation of circulating triglyceride levels through the upregulation of Angptl8. In our data, the loss of GNMT resulted in the increase of Angptl8 expression and Akt phosphorylation in primary hepatocytes isolated from GNMT-KO mice. The blockage of Akt phosphorylation by the PI3K inhibitor or the Akt inhibitor inhibited Angptl8 expression in the primary GNMT-KO hepatocytes. Since Angptl8 is a lipid metabolism-associated hormone that is released mainly from liver and adipose tissue, our study provides the possibility that HFD-induced downregulation of GNMT may increase the production and release of Angptl8, leading to the increase of plasma triglyceride levels in metabolic syndrome.

For its enzymatic function, GNMT is involved in transcriptional regulation of genes by inhibiting the conversion of SAM, leading to the alterations of genomic DNA methylation pattern. In addition, GNMT has been linked to detoxification and anti-oxidation pathways in HCC [47]. Although the molecular mechanism is not clear, phosphorylation of GNMT at Serine 9 residue leads to its nuclear translocation, which promotes the expression of genes with a detoxification function [48]. In the central nervous system, systematic knockout of GNMT resulted in the impairment of learning and memory by diminishing neurogenesis through the excess SAM-induced alteration of the bFGF-stimulated MAP kinase signaling cascade [49]. Recently, GNMT has been considered as an adaptor protein that binds to signaling molecules independent of its enzyme activity [30]. GNMT interacts with PREX2, a novel PTEN inhibitor [31], leading to the degradation of PREX2 through an E3 ligase HectH9-mediated proteasomal ubiquitination pathway. The GNMT-PREX2 interaction causes the increase of PTEN activity and the decrease of Akt phosphorylation as a consequence. In our study, we found that the loss of GNMT resulted in the increase of Akt phosphorylation in primary GNMT-KO hepatocytes and an elevation of Angptl8 expression. Our data indicated that the GNMT expression was associated with Angptl8 expression. Indeed, in our HFD-fed mice and from a microarray dataset analysis, GNMT protein and mRNA levels were inversely correlated with Angptl8 mRNA and protein expression. This association of GNMT and Angptl8 may be due to the inhibitory function of GNMT on the insulin-induced Akt signaling pathway. However, more experiments are required to explore the detail mechanism of GNMT-regulated Angptl8 expression.

In conclusion, our study suggests that downregulation of hepatic GNMT and upregulation of hepatic and circulating Angptl8 may be a potential biomarker for HFD-induced metabolic syndrome. The inverse correlation of GNMT and Angptl8 expression may be through the inhibitory function of GNMT on insulin-induced Akt phosphorylation. Since GNMT and Angptl8 play important role in the homeostasis of lipid metabolism, targeting GNMT and/or Angptl8 may be a potential therapeutic strategy to overcome diet-induced metabolism syndrome.

## 4. Materials and Methods 

### 4.1. Animal Study

Twenty age-matched C57BL/6J mice (6 weeks old; male; Charles River. Technology, BioLASCO Taiwan Co, Ltd., Taipei, Taiwan) were randomly divided into ND-fed and HFD-fed groups with 10 mice each. There was no significant difference in initial body weight among the two groups (mean body weight of ND group = 18.36 ± 0.41 g, mean body weight of HFD group = 18.70 ± 0.40 g, *p* = 0.61). The mice were fed with a control diet (catalog number: D12450H, 10 kcal% fat, Research Diets, New Brunswick, NJ, USA) or a high-fat diet (catalog number: D12451, 45 kcal% fat, Research Diets) for 12 weeks. Body weight gain and feed consumption were monitored weekly to determine the feed efficiency ratio (FER). Body mass index (BMI) was calculated according to the relationship between body weight and naso-anal length squared. After the end point of experiment, the mice received a 12 h fasting and were anesthetized. The blood samples were collected by cardiac puncture, centrifuged at 800 *g* for 10 min at 4 °C, and the serum was stored at −80 °C. The livers were collected, weighted, and stored at −80 °C. The GNMT-KO mice in C57BL/6 background [50] and their littermate wild-type control were generated and maintained in specific pathogen-free conditions in accordance with the regulations at the Animal Center, Kaohsiung Medical University. All the animal experiments were performed in accordance with a protocol approved by the Institutional Animal Care and Use Committee of Kaohsiung Medical University (IACUC code: 102186; 25 Dec 2013).

### 4.2. Biochemical Analysis and ELISA

The serum glucose concentrations and the levels of total cholesterol and triglycerides were measured using the Fuji DRI-CHEM NX500V system (Fuji Film, Tokyo, Japan). The plasma insulin levels were determined using a RayBio mouse insulin ELISA kit (catalog number: ELM-Insulin, RayBiotech, Norcross, GA, USA). The plasma Angptl8 levels were measured using a mouse Betatrophin ELISA kit (catalog number: E11633m, Wuhan EIAab Science Co, Ltd., Wuhan, China). The absorbance measurement was performed using a Microplate Spectrophotometer (Epoch, BioTek, Winooski, VT, USA) at 450 nm wavelength.

### 4.3. Primary Hepatocyte Culture

Primary mouse hepatocytes were isolated from adult male C57BL/6J and GNMT-KO mice by a two-step collagenase perfusion method with modifications [51]. Briefly, the livers were perfused via the inferior vena cava with pre-warmed Ca^2+^-free Hank’s Balanced Salt Solution (HBSS) containing 0.1 mM EGTA and heparin (2 U/mL), followed by perfusion with Dulbecco’s Modified Eagle’s medium (DMEM) containing collagenase type IV (100 U/mL, catalog number: C5138, Sigma-Aldrich, St. Louis, MO, USA) and 0.5% fetal bovine serum (FBS) (Gibco, Thermo Fisher Scientific, Waltham, MA, USA). Hepatocytes were separated from non-parenchymal cells by low-speed centrifugation (50 *g*, 5 min, twice) at 4 °C. Hepatocytes were plated on type I collagen (catalog number: C3867, Sigma-Aldrich, st. Louis, MO, USA)-coated 6 cm petri dishes containing DMEM supplemented with 10% FBS, 4 μg/mL insulin, 1 μM dexamethasone, 1% penicillin/streptomycin, 15 mM hydroxyethyl-piperazineethane-sulfonic acid (HEPES) buffer, and 2 mM L-glutamine at a density of 2 × 10^5^ cells/dish. The culture medium was replaced with fresh medium every day and the primary cultures were maintained at 37 °C in a humidified chamber with 5% CO_2_.

### 4.4. Plasmids and Transfection

The dnAkt and myrAkt plasmids were kind gifts from Dr. Pei-Jung Lu, Institute of Clinical Medicine, National Cheng Kung University, Tainan, Taiwan. A K179M kinase-dead form of Akt1 (dnAkt), and the N-terminally myristoylation signal (MGSSKSKPK)-attached Akt (myrAkt) were subcloned into a pcDNA3.1 vector [52,53]. For transient expression, the primary hepatocytes were seeded in 6 cm petri dishes and grown to 80% confluence. The transfection was performed using a Lipofectmine 2000 (Invitrogen Life Technologies, Thermo Fisher Scientific, Waltham, MA, USA) and 4 μg plasmid DNA in OPTI-MEM medium (Invitrogen Life Technologies). Four hours after transfection, the OPTI-MEM medium was replaced by fresh DMEM supplemented with 10% FBS, 4 μg/mL insulin, 1 μM dexamethasone, 1% penicillin/streptomycin, 15 mM HEPES, and 2 mM l-glutamine.

### 4.5. Western Blot

For the Western blot analysis, the liver tissues or primary hepatocytes were lysed in 1× radioimmunoprecipitation assay (RIPA) buffer containing protease inhibitor cocktails (Roche Diagnostic GmbH, Mannheim, Germany). Protein concentration was determined using a Bio-Rad DC protein assay kit (Bio-Rad, Hercules, CA, USA). The cell lysate was loaded onto a 10% Sodium dodecyl sulfate (SDS)-polyacrylamide gel for electrophoresis and then electrotransferred onto polyvinylidene difluoride membranes. The membranes were incubated with the indicated primary antibodies, followed by horseradish peroxidase-conjugated secondary antibodies and an enhanced chemiluminescence solution (NEN Life Science, Boston, MA, USA). The following antibodies were used for the experiments: Anti-GNMT (catalog number: 14-1, YMAC Bio Tech, Taipei, Taiwan), anti-Akt (catalog number: #4685), anti-phospho-Akt T308 (catalog number: #13038), anti-phosphoAkt S473 (catalog number: #9271), anti-ERK1/2 (catalog number: #4695), anti-phosphoERK1/2 (catalog number: #4376) (Cell Signaling Technology, Danvers, MA, USA), anti-Angptl8 (catalog number: TA326696) (Origene, Rockville, MD, USA), and anti-Actin (Sigma-Aldrich) antibodies.

### 4.6. Quantitative Real-Time PCR

Total RNA was extracted from liver tissues and primary hepatocytes using TRIzol reagent (Invitrogen, Carlsbad, CA, USA). cDNA was synthesized using an MMLV Kit (Invitrogen) according to the manufacturer’s instructions. Quantitative real-time PCR (qPCR) was performed using the Fast SYBR Green Master Mix and run on a StepOnePlus real-time PCR system (Invitrogen) with a reaction program as follows: A total of 20 s at 95 °C, followed by 40 cycles at 95 °C for 3 s and annealing at 60 °C for 30 s. The mRNA expression of the indicated genes was determined using the 2^−ΔΔ*C*t^ method and the data were normalized to those of the housekeeping gene glyceraldehyde-3-phosphate dehydrogenase (GAPDH). The specific primer sequences of the tested genes are listed as follows: mAngptl8, forward: CCAGTTGTGCTGCAAGGAAC, reverse: TTGCTTCTGTCTCCGCTCTG; mGNMT, forward: GTTGACGCTGGACAAAGA, reverse: AGCCTGTGCTGAGGATA; mGAPDH, forward: GGCAAATTCAACGGCACA, reverse: GTTAGTGGGGTCTCGCTCTG.

### 4.7. Statistical Analysis

All the observations were confirmed by at least three independent experiments. The results are presented as the mean ± standard deviation. We used two-tailed paired Student’s *t*-tests for all the pair-wise comparisons. Comparisons between multiple groups were undertaken using one-way ANOVA followed by Dunnett’s test. In all the comparisons and differences were considered statistically significant at *p* < 0.05.

## Figures and Tables

**Table 1:** Effects of high fat-diet on biometric variables and consumption in C57BL/6J mice.

Groups	ND	HFD
Food consumption (g/week)	19.74 ± 2.17	17.17 ± 1.92
Weight gain (g)	2.71 ± 0.35	5.18 ± 0.79 *
FER (%)	8.01 ± 0.87	17.60 ± 1.35 *
BMI (g.cm^−2^)	0.30 ± 0.01	0.32 ± 0.02 *
Liver weight (g)	1.02 ± 0.10	1.93 ± 0.29 *
HSI (%)	4.74 ± 0.45	7.87 ± 0.64 *

BMI, body mass index; FER, feed efficiency ratio; HSI, hepatosomatic index; ND, normal diet; HFD, high fat diet; * *p* < 0.05.

## Abbreviations

BMI	Body mass index
FER	Food efficiency ratio
GNMT	Glycine *N*-methyltransferase
HFD	High fat diet
HSI	Hepatosomatic index
ND	Normal diet
SAM	s-adenosylmethionine
SAH	s-adenosylhomocysteine
TG	Triglyceride
